# Nitrogen Metabolism Genes from Temperate Marine Sediments

**DOI:** 10.1007/s10126-017-9741-0

**Published:** 2017-03-10

**Authors:** Carolina Reyes, Dominik Schneider, Marko Lipka, Andrea Thürmer, Michael E. Böttcher, Michael W. Friedrich

**Affiliations:** 1 Microbial Ecophysiology, University of Bremen, Leobener Strasse, D-28359 Bremen, Germany; 2 Department of Environmental Geosciences, University of Vienna, Althanstrasse 14, 1090 Vienna, Austria; 3 Department of Genomic and Applied Microbiology, University of Göttingen, Grisebachstrasse 8, D-37077 Göttingen, Germany; 4 Geochemistry and Stable Isotope Biogeochemistry Group, Leibniz Institute for Baltic Sea Research (IOW), Seestrasse 15, D-18119 Warnemünde, Germany

**Keywords:** Nitrogen, Metagenome, Marine, Sediments

## Abstract

In this study, we analysed metagenomes along with biogeochemical profiles from Skagerrak (SK) and Bothnian Bay (BB) sediments, to trace the prevailing nitrogen pathways. NO_3_^−^ was present in the top 5 cm below the sediment-water interface at both sites. NH_4_^+^ increased with depth below 5 cm where it overlapped with the NO_3_^−^ zone. Steady-state modelling of NO_3_^−^ and NH_4_^+^ porewater profiles indicates zones of net nitrogen species transformations. Bacterial protease and hydratase genes appeared to make up the bulk of total ammonification genes. Genes involved in ammonia oxidation (*amo*, *hao*), denitrification (*nir*, *nor*), dissimilatory NO_3_^−^ reduction to NH_4_^+^ (*nfr* and *otr*) and in both of the latter two pathways (*nar*, *nap*) were also present. Results show ammonia-oxidizing bacteria (AOB) and ammonia-oxidizing archaea (AOA) are similarly abundant in both sediments. Also, denitrification genes appeared more abundant than DNRA genes. 16S rRNA gene analysis showed that the relative abundance of the nitrifying group *Nitrosopumilales* and other groups involved in nitrification and denitrification (*Nitrobacter*, *Nitrosomonas*, *Nitrospira*, *Nitrosococcus and Nitrosomonas*) appeared less abundant in SK sediments compared to BB sediments. *Beggiatoa* and *Thiothrix* 16S rRNA genes were also present, suggesting chemolithoautotrophic NO_3_^−^ reduction to NO_2_^−^ or NH_4_^+^ as a possible pathway. Our results show the metabolic potential for ammonification, nitrification, DNRA and denitrification activities in North Sea and Baltic Sea sediments.

**Electronic supplementary material:**

The online version of this article (doi:10.1007/s10126-017-9741-0) contains supplementary material, which is available to authorized users.

## Introduction

**Fig. 1:**
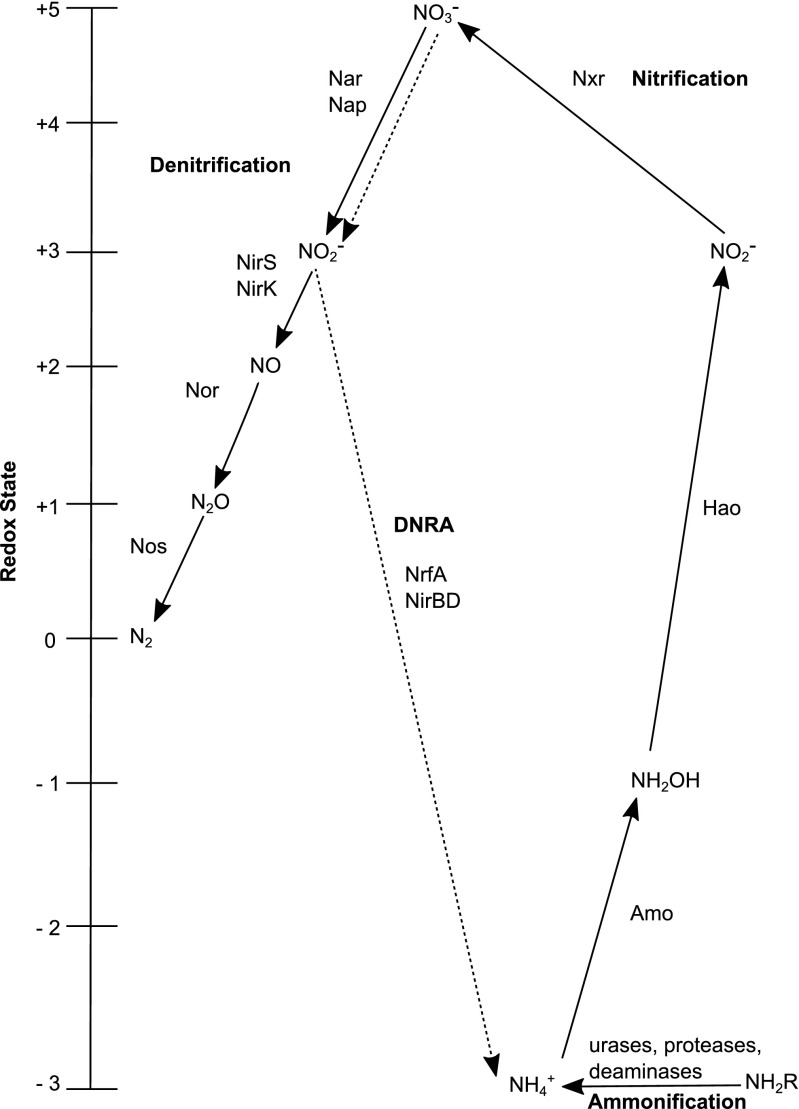
Ammonification, DNRA, nitrification and denitrification pathways and the enzymes involved in those pathways (Figure modified after Cabello et al. (2009)

Nitrogen is one of the essential elements of life and its cycle is driven mostly by microbial activities. Anthropogenic and physical processes also contribute to different sources of nitrogen (Delwiche 1970; Söderlund and Svensson 1976). Elucidating the pathways of nitrification and denitrification has been a topic of interest since the late nineteenth century when Winogradsky isolated *Nitrosococcus Winogradsky* (Winogradsky 1890). In marine sediments, nitrogen is cycled as part of the redox zonation in the suboxic zone (Froelich et al. 1979; Jørgensen 1989; Middelburg et al. 1993; Herbert 1999). Ammonium (NH_4_^+^) is mainly liberated into the porewater by ammonification involving multiple steps of microbial breakdown of proteins, peptides and amino acids by proteolytic enzymes and deaminases (Herbert 1999 and references therein; Fig. 1). Also, the breakdown of nucleic acids can lead to release of urea (Therkildsen et al. 1996, 1997). Whether certain steps in the ammonification process in marine sediments dominate more than others remain poorly understood.

With respect to nitrification, whereby ammonia (NH_4_^+^/NH_3_) is oxidized to NO_3_^−^, it is still unclear whether ammonia-oxidizing archaea (AOA) (Könneke et al. 2005; Wuchter et al. 2006; Francis et al. 2007) or ammonia-oxidizing bacteria (AOB) (Freitag et al. 2006) play a more important role in the first step of this pathway in marine sediments (Schleper and Nicol 2010). AOA (e.g. *Nitrosopumilus*) and AOB (e.g. *Nitrosospira*, *Nitrosococcus*, *Nitrosomonas*) mediate oxidation of NH_3_ to nitrite (NO_2_^−^) using the copper-containing enzyme ammonia monooxygenase (AMO). While AOB use the protein hydroxylamine oxidoreductase (HAO) as an intermediate in the ammonia oxidation process, it is not yet clear what that intermediate protein is in the AOA process (Norton et al. 2001; Walker et al. 2010; Fig. 1). In the Baltic Sea, AOA have been observed to be abundant in the water column (Pitcher et al. 2011; Bale et al. 2013) and in sediments (Jørgensen 1989). It also remains debatable which groups of nitrate-oxidizing bacteria (NOB) including *Nitrobacter*, *Nitrospira*, and *Nitrospina* (Teske et al. 1994; Spieck and Bock 2005; Lücker et al. 2010) are likely to contribute most to oxidation of NO_2_^−^ to NO_3_^−^ in marine sediments.

Besides ammonification, dissimilatory nitrate (NO_3_^−^) reduction to NH_4_^+^ (DNRA) can also serve as a source of NH_4_+ for primary producers and benthic organisms, yet it remains an understudied pathway (Giblin et al. 2013). In this step, respiratory and fermentative microorganisms reduce NO_3_^−^ to NH_4_^+^ (Fig. 1). In the first step of denitrification, NO_3_^−^ is reduced to NO_2_^−^ (Nar proteins). Once NO_2_^−^ is produced, it can proceed to the next step of the denitrification pathway, or NO_2_^−^ can be converted to NH_4_^+^ (via NrfA, NirBD proteins) via the DNRA pathway (Fig. 1). A number of bacteria including *Beggiatoa* are capable of DNRA (Baggs and Phillipot 2011; Giblin et al. 2013).

In comparison to DNRA, the denitrification pathway is a more intensively studied pathway in marine sediments. Denitrification includes the conversion of NO_3_^−^ to NO_2_^−^, NO_2_^−^ to nitric oxide (NO) (Nir proteins), NO to nitrous oxide (N_2_O) (Nor proteins) and N_2_O to N_2_ (Nos proteins) (Teske et al. 1994; Spieck and Bock 2005; Lücker et al. 2010; Pauleta et al. 2013 and references therein). Bacteria that are capable of completing different parts of the reaction are phylogenetically diverse but most are members of the *Proteobacteria* and are facultative aerobes (Shapleigh 2013). Under low oxygen conditions, most AOB can reduce NO_3_^−^ to N_2_O (Fig. 1; Ward 2013). AOB such as *Nitrobacter* and *Nitrosomonas* can reduce NO_3_^−^ to NO_2_^−^ using bacterial NO_3_^−^ reductases (Nar; Lücker et al. 2010) (Fig. 1). *Nitrosomonas* have also been shown to reduce NO_2_^−^ to NO using the copper-containing enzyme NirK and associated Nir proteins (Cantera and Stein 2007) and to reduce NO to N_2_O with the Nor proteins (Spieck and Bock 2005) (Fig. 1). Furthermore, several *Thiothrix* spp. have been shown to reduce NO_3_^−^ to NO_2_^−^ by oxidizing thiosulphate using the *narGHI* gene products (Trubitsyn et al. 2013). In spite of our better knowledge about the denitrification pathway, the environmental factors that determine the balance between DNRA and denitrification are far from being understood. In some estuary sediments, DNRA has been found to be the dominant process influencing the fate of NO_3_^−^ as opposed to denitrification (Soonmo and Gardner 2002; Giblin et al. 2010). Continuing to determine the abundances of microbial groups throughout the Baltic Sea and other areas could help elucidate their level of importance with respect to nitrogen cycling in these areas.

A number of studies have identified AOA and AOB in the water column and sediments using primers for 16S rRNA and the *amoA* gene via cloning and sequencing or by using lipid biomarker techniques (Francis et al. 2005, 2007; Dang et al. 2008; Pitcher et al. 2011, Bale et al. 2013). Similar techniques have been applied to study denitrifying communities in marine sediments (Scala and Kerkhof 1998, 1999; Braker et al. 2000; Michotey et al. 2000). Furthermore, metagenomics has been extensively used as an initial step in inferring the functional and metabolic potential of microbial communities in environmental studies (Xie et al. 2011; Dini Anderote et al. 2012; Kimes et al. 2013; Scott et al. 2014). Thureborn et al. (2013) studied metagenomes associated with nitrification and denitrification in three anoxic sediment samples from Landsort Deep (Baltic Sea). However, a combined metagenomic and biogeochemical approach to study ammonification, nitrification, DNRA and denitrification pathways has not been reported for suboxic surface sediments of the North Sea and Baltic Sea.

To better understand which microorganisms and metabolic pathways could be contributing most to nitrification, ammonification, DNRA and denitrification, we studied N-cycling gene distributions in three sediment samples using an Illumina technique. Sediments came from the Skagerrak (SK), located at the North Sea-Baltic Sea, and from the Bothnian Bay (BB) located in the Baltic Sea. Computational analysis of the sequencing data using the Metagenomic RAST Analysis Server (MG-RAST) pipeline allowed for the analysis of an unprecedented number of sequences in these sediments providing a more complete representation of microbial communities and their genes. Porewater concentrations of SK and BB ferruginous sediments showed on-going NO_3_^−^ reduction and NH_4_^+^ production providing the opportunity to relate these pathways to biogeochemical zones in the surface sediments.

## Methods

### Study Site

**Fig. 2:**
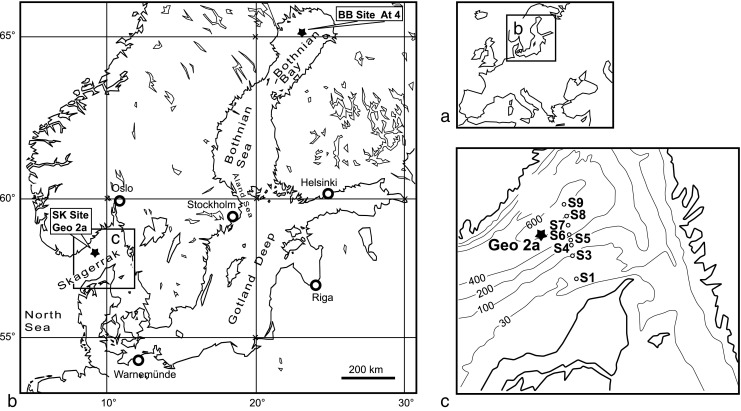
(**a**) Map of the Baltic Sea (**b**) showing locations of the two sampling Sites Geo 2a and At4. **c** The Skagerrak showing the locations of Site Geo2a with respect to the locations of Sites S1 through S9 of the transect studied by Canfield et al. (1993)

Sediment cores of up to 38 cm in length (10 cm in diameter) were taken during the RV Meteor cruise No. M86-1 in November 2011 using a multi-corer device (Oktopus GmbH, Kiel, Germany). The two sampling locations considered in this study were BB site At4 (65° 26.71′ N/23° 17.92′ E) and SK Site Geo 2a (58° 29.513′ N/9° 35.855′ E) (Fig. 2). Samples were taken from a water depth of 75 m at site At4 and from a water depth of 554 m at site Geo 2a. After collection, cores for microbiological analyses from each site were frozen at −20 °C on board the ship and later transferred to −80 °C in the shore-based laboratory. For a more detailed description of the study sites refer to Reyes et al. (2016).

### Porewater Analysis

Parallel cores were recovered for geochemical analyses and porewater was extracted from 23 depths from SK cores and 16 depths from BB cores directly after recovery by using Rhizon samplers (Rhizosphere Research Products B.V, Wageningen, The Netherlands) (Seeberg-Elverfeldt et al. 2005). Porewater samples for NO_3_^−^ and NH_4_^+^ were kept frozen until later analysis at the IOW (Leibniz Institute for Baltic Sea Research Warnemünde) and measured after the methods of Grasshoff et al. (1999) by using a continuous flow nutrient analyser (QuAAtro, Seal Analytical GmbH, Norderstedt, Germany). Additional geochemical measurements of these cores were made and are discussed in Reyes et al. (2016).

### Pore Water Modelling

Interpretation of interstitial water profiles of dissolved NO_3_^−^ and NH_4_^+^ at sites Geo 2a and At4 were carried out using the PROFILE (Berg et al. 1998) and the REC (Lettmann et al. 2012) models, considering steady-state conditions. Porewater profiles for the dissolved species used in the modelling can be found in Reyes et al. (2016). Local or non-local irrigation was neglected in the interpretation of porewater profiles. The diffusion coefficients in free solution at in situ salinity and temperature were calculated according to Boudreau (1997) and Schulz and Zabel (2006). The molecular diffusivity in the sediment was corrected for tortuosity according to Iversen and Jørgensen (1993), considering sediment porosities after Flemming and Delafontaine (2000). Both models calculate net and not gross process rates.

### DNA Extraction

Frozen cores were sliced into 1 cm or 2 cm diameter discs with a band saw (K330S, Paul Kolbe GmbH, Elchingen, Germany) with a WIKUS blade (WIKUS DIAGRIT S Nr. 572 D254 VA, WIKUS-Sägenfabrik, Spagenburg, Germany). The blade was cleaned and sterilized with ethanol (70%) after cutting each slice. The sediment that was in contact with the blade was removed, and only the interior parts of the frozen disc were sectioned into aliquots for DNA extraction. DNA was extracted from two BB samples (BB 3–4 cm and BB 6–7 cm) and one SK sample (SK 6–8 cm). Extractions were made from ~0.5–0.6 g of sediment based on the method of Lueders et al. (2004). Following extraction, samples were pooled and the nucleic acid pellet was dissolved in 200 μl of RNase/DNase free water. Humic substances and phenols from the crude extracts were removed with the Zymo PCR Inhibitor Removal kit (Zymo Research, Freiburg, Germany) following the manufacturer instructions. The total DNA was purified by digesting the RNA with 10 μl of 10 mg/ml of Roche RNase A (Sigma-Aldrich, Munich, Germany) for 5 min at room temperature and the Zymo Genomic DNA Clean and Concentrator kit (Zymo Research, Freiburg, Germany). The following modification was made to the manufacturer method: following the first elution through the column, the eluate was not discarded. Instead the eluate was added to a new column. Both columns were processed according to manufacturer instructions and total DNA eluted with water from both columns. Extracts were checked for nucleic acid concentration by NanoDrop Spectrophotometer ND-1000 (PeqLab-VWR International GmbH, Erlangen, Germany) and using the Invitrogen Quanti-iT PicoGreen dsDNA Assay Kit (Thermo Fisher Scientific, Darmstadt, Germany) following the manufacturer instructions. Fluorescence measurements were made using a fluorimeter (Fluoroskan Ascent FC, Thermo Labsystems, Milford, USA).

### Metagenome Sequencing

DNA shotgun libraries were generated using the Nextera DNA Library preparation kit following the manufacturer instructions (Illumina, San Diego, USA). The metagenomes were sequenced in a 112-bp paired end single indexed run with the Genome Analyzer IIx (Illumina, San Diego, USA).

### Processing of Short Reads

Quality filtering of the metagenomic reads was performed with trimmomatic (0.25) (Bolger et al. 2014). This included the removal of Illumina adaptors and sequences that were shorter than 50 bp. Additionally, sliding window clipping was performed to remove read spaces that had an average quality below 15 (Phred score 33) within a 4-bp window. Samples were analysed using the Metagenomic Analysis Server (MG-RAST) (Meyer et al. 2008) using default settings. Sequences were normalized via transformation, standardization and multiple sample scaling as described in the MG-RAST manual (Wilke et al. 2015). Briefly, for normalization, raw abundances were transformed by a (log2 + 1) transformation, standardized and scaled from 0 to 1 (Meyer et al. 2008).

To compare 16S rRNA sequence abundances, the MG-RAST M5RNA database, which integrates SILVA, Greengenes and RDP databases, was used. An *e* value cut-off of 1e-05, minimum identity 60% cut-off and a minimum alignment cut-off of 15 were the default parameters used in the analysis. The best hit classification option was selected. Before analysis, BB forward and reverse sequences were clustered as one group and SK forward and reverse sequences as another group using the PCoA option. Following classification, the average of forward and reverse abundances was compared between samples. Normalized abundances of AOA were combined and compared to normalized abundances of AOB.

Protein-coding genes were annotated against the SEED Level (function) Subsystems of MG-RAST using the default parameters described above. The hierarchical classification option was selected. Ward Clustering and Bray Curtis distance options were selected for the heat map analysis. Normalization and standardization of forward and reverse sequences was accomplished using the R statistical computing system (version 3.2.1; R Core Team 2013), R package “matR” (version 0.9) (Braithwaite and Keegan 2013) and accessory apps built on the “matR” package for MG-RAST (Keegan 2015). Following classification, the average of forward and reverse abundances were compared between samples.

A one-way ANOVA (alpha 0.05) and unpaired *t* tests (alpha 0.05) were used to determine if the abundances of protein-coding genes between and within samples were significant (StatPlus, Microsoft Excel 2011). When comparing between sites, BB samples were grouped together for the analysis. For the ANOVA analysis, the following gene abundances were compared: SK DNRA, SK denitrification, SK both (referring to genes belonging to both DNRA and denitrification pathways), BB DNRA, BB denitrification and BB both. To determine differences in gene abundances between sites in terms of ammonification, DNRA, denitrification and genes belonging to both DNRA and denitrification pathways, the following steps were carried out: (i) an *F* test was used to determine equal or unequal variance, (ii) differences in the number of genes being compared between sites was corrected by randomly sampling the BB gene abundance data to match the number of genes in the SK and (iii) a two-tailed distribution was used to determine significance. When comparing between BB34 and BB67 samples, BB AOA vs. BB AOB groups and SK AOA vs. SK AOB groups, step (ii) was excluded because the number of genes were similar between these samples or groups.

## Results

### Characteristics of Skagerrak and Bothnian Bay Metagenomes

Following Illumina sequencing of two BB samples and one SK sample, approximately 4.9 Gb of sequencing data were generated consisting of 19–21 million (BB samples) and 4 million (SK sample) reads with an average read size of 112 bp. The metagenome data was deposited in the MG-RAST database (Table S1). To our knowledge, this is the largest metagenomic dataset to date of Baltic Sea sediments. An average of 700–800 k genes were annotated as non-rRNA proteins for BB samples and 155 k genes for the SK sample. For comparative purposes, reads were normalized to rule out the effect of inter-sample variations on the read abundances as described in the “Methods” section above.

### Overview of Nitrogen Metabolism Genes Detected in Metagenomes

**Fig. 3:**
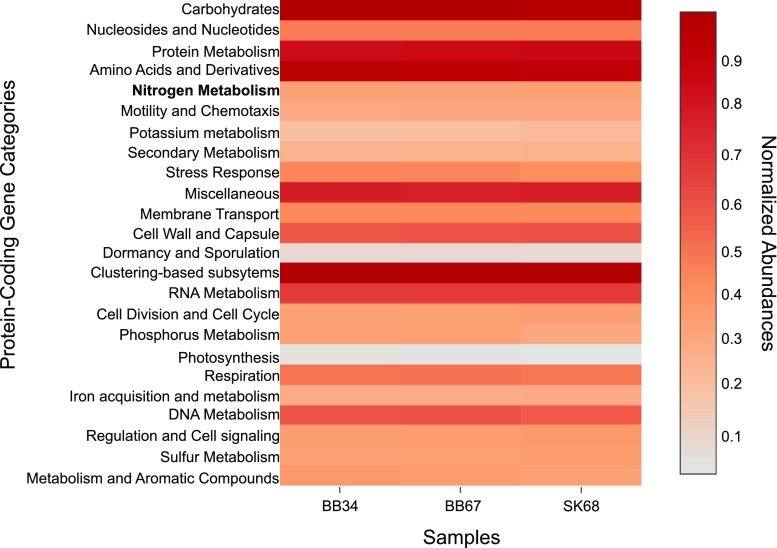
Heat map showing normalized protein-coding gene abundances predicted to be involved in various metabolic pathways detected in SK and BB metagenomes. The most abundant genes (*red*) and least abundant genes (*grey*). BB34 refers to sample 3–4 cmbsf (centimetre below sea-floor), BB67 refers to sample 6–7 cmbsf and SK68 refers to sample 6–8 cmbsf. Values for samples were scaled from 0 (minimum value) to 1 (maximum value) using a uniform scaling method implemented in the MG-RAST pipeline. Forward and reverse abundances were averaged and a single value is reported per sample

**Fig. 4:**
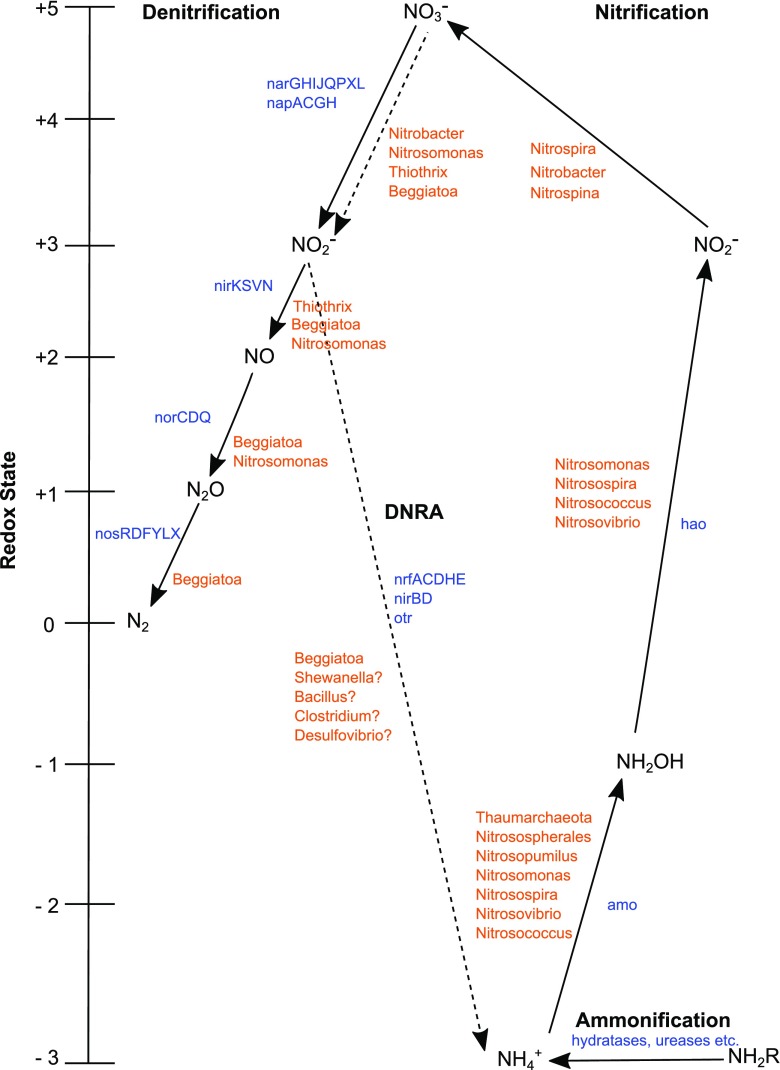
Ammonification, DNRA, nitrification and denitrification pathways based on protein-coding genes detected in SK and BB samples. Enzymes (*blue*) and taxa (*orange*) that could potentially be involved in each pathway are written next to each arrow. *Question marks* indicate uncertainty in the organism’s involvement in the pathway despite detection. Figure modified after Cabello et al. (2009)

Protein-coding genes involved in the nitrogen cycle detected in our samples were not the most abundant of all genes in samples (Fig. 3). Furthermore, genes for ammonification, DNRA, nitrification and denitrification pathways were detected in metagenomes and are summarized in Fig. 4. One-way ANOVA analysis of DNRA, denitrification gene abundances and genes involved in both pathways revealed a significant difference between samples (*P* < 0.05).

### Biogeochemistry in Surface Sediments

**Fig. 5:**
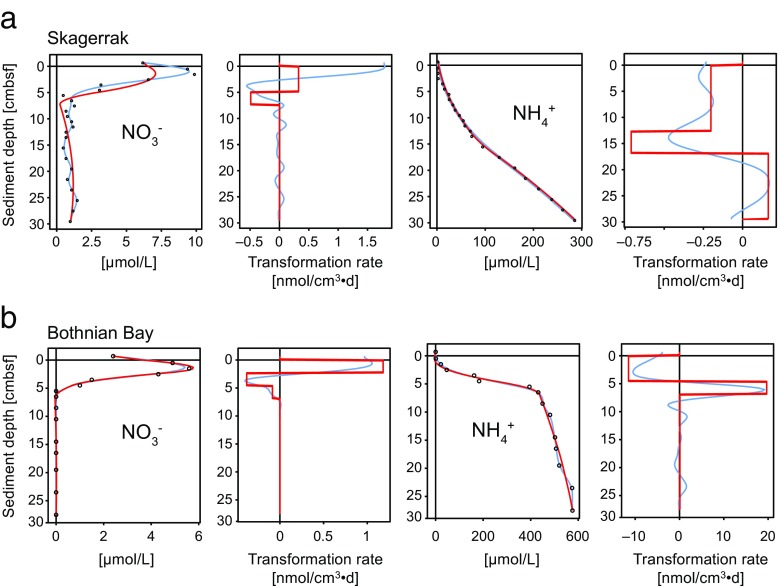
Model based on porewater concentrations showing predicted rates of NO_3_^−^ and NH_4_^+^ production and consumption in sediments. Porewater concentrations and predicted rates of NO_3_^−^ production and consumption in SK samples (**a**) and in BB samples (**b**). *Red lines* (PROFILE model), *blue lines* (REC model)

The vertical profiles of dissolved NO_3_^−^ and NH_4_^+^ observed at SK (Fig. 5a) and BB (Fig. 5b) indicate different zones of net production or consumption. They follow the expected porewater redox zonation for sediments (e.g. Froelich et al. 1979). Based on steady-state modelling (Berg et al. 1998; Lettmann et al. 2012), the gradients of measured dissolved NO_3_^−^ and NH_4_^+^ were interpreted quantitatively to estimate the zones of net (de-) nitrification and ammonium production. NO_3_^−^ and NH_4_^+^ models for SK (Fig. 5a) and for BB (Fig. 5b) yield similar rates and vertical zones of net transformations, with the major difference being the use of discrete zones in the PROFILE model (Berg et al. 1998), whereas continuous smooth rate changes are used in the REC model (Lettmann et al. 2012).

At the SK site, NO_3_^−^ occurs below 0.5 cm and is present down to about 5 cm (Fig. 5a). Calculated rates of NO_3_^−^ consumption from concentration gradients show a maximum consumption at 5 cm (Fig. 5a) due to NO_3_^−^ reduction. NH_4_^+^ occurs below 2.5 cm within the zone of NO_3_^−^ reduction and increases constantly below this depth (Fig. 5a). The rate calculations indicate net NH_4_^+^ consumption occurs below the surface and a second consumption peak below ~12 cm, and net production below ~17 cm (Fig. 5a).

At the BB site, NO_3_^−^ increases immediately below the surface and is present to 5 cm (Fig. 5b). Modelling results indicate NO_3_^−^ production in the top ~4 cm (Fig. 5b). Nitrification at the sediment-water interface is the likely source of NO_3_^−^. Below 4 cm, NO_3_^−^ is consumed by NO_3_^−^ reduction. NH_4_^+^ appears at 0.5 cm and increases strongly below 5 cm but only a minor increase occurs below this depth (Fig. 5b). Modelling results indicate NH_4_^+^ production mainly between 5 and 10 cm, while its production is masked by NH_4_^+^ consumption by nitrification in the top 4 cm (Fig. 5b).

### Ammonification

**Fig. 6:**
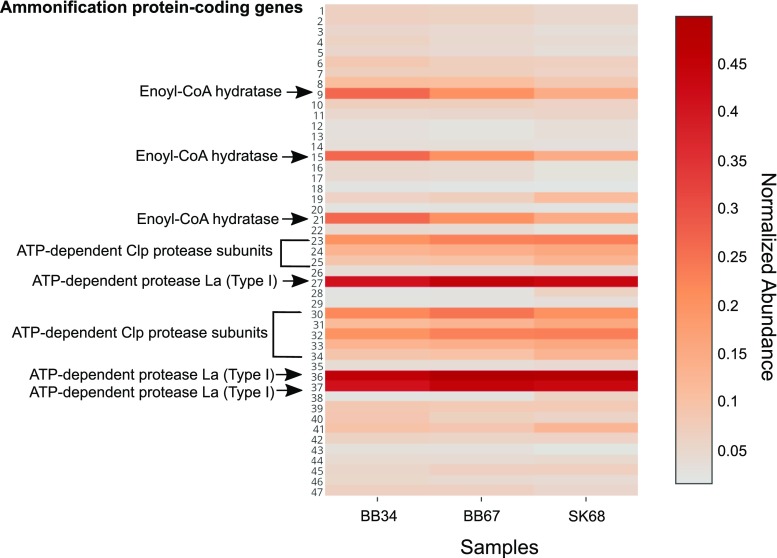
Heat map showing normalized protein-coding gene abundances predicted to be involved in ammonification metabolism detected in SK and BB metagenomes. The most abundant genes (*red*) and least abundant genes (*grey*). BB34 refers to sample 3–4 cmbsf (centimetre below sea-floor), BB67 refers to sample 6–7 cmbsf and SK68 refers to sample 6–8 cmbsf). *Numbers on the y-axis* correspond to the genes listed in Table 1. Values for samples were scaled from 0 (minimum value) to 1 (maximum value) using a uniform scaling method implemented in the MG-RAST pipeline. Forward and reverse abundances were averaged and a single value is reported per sample

The relative abundance of protein-coding genes involved in ammonification appeared abundant in SK and BB samples (Table 1). However, an unpaired *t* test analysis did not reveal any significant difference in ammonification gene abundances between samples (*P* > 0.05). Ammonification genes were composed of protease, hydratase, peptidase, urease and deaminase genes (Fig. 6).

**Table 1:** Protein-coding genes potentially involved in ammonification in SK and BB samples. Normalized abundances are shown in Fig. 6

No.	Protein-coding category
1	Arginine_and_Ornithine_Degradation----N-carbamoylputrescine amidase (3.5.1.53)
2	Polyamine_Metabolism----N-carbamoylputrescine amidase (3.5.1.53)
3	Branched_chain_amino_acid_degradation_regulons----Hydroxymethylglutaryl-CoA lyase (EC 4.1.3.4)
4	Histidine_Degradation----Histidine ammonia-lyase (EC 4.3.1.3)
5	Leucine_Degradation_and_HMG-CoA_Metabolism----Hydroxymethylglutaryl-CoA lyase (EC 4.1.3.4)
6	Methionine_Degradation----Cystathionine gamma-lyase (EC 4.4.1.1)
**7**	Branched_chain_amino_acid_degradation_regulons----Enoyl-CoA hydratase [isoleucine degradation] (EC 4.2.1.17)
8	Histidine_Degradation----Urocanate hydratase (EC 4.2.1.49)
**9**	Isoleucine_degradation----Enoyl-CoA hydratase (EC 4.2.1.17)
10	Isoleucine_degradation----Enoyl-CoA hydratase [isoleucine degradation] (EC 4.2.1.17)
11	Phenylalanine_and_Tyrosine_Branches_from_Chorismate----Prephenate dehydratase (EC 4.2.1.51)
12	Threonine_anaerobic_catabolism_gene_cluster----Threonine dehydratase, catabolic (EC 4.3.1.19)
13	Threonine_degradation----Threonine dehydratase, catabolic (EC 4.3.1.19)
14	Threonine_degradation----Threonine dehydratase (EC 4.3.1.19)
**15**	Valine_degradation----Enoyl-CoA hydratase (EC 4.2.1.17)
16	Urea_decomposition----Urease alpha subunit (EC 3.5.1.5)
17	Urease_subunits----Urease alpha subunit (EC 3.5.1.5)
18	Inositol_catabolism----Epi-inositol hydrolase (EC 3.7.1.-)
19	Predicted_carbohydrate_hydrolases----COG2152 predicted glycoside hydrolase
20	Acetyl-CoA_fermentation_to_Butyrate----3-hydroxybutyryl-CoA dehydratase (EC 4.2.1.55)
**21**	Acetyl-CoA_fermentation_to_Butyrate----Enoyl-CoA hydratase (EC 4.2.1.17)
22	Novel_non-oxidative_pathway_of_Uracil_catabolism----Urease alpha subunit (EC 3.5.1.5)
23	Proteasome_bacterial----ATP-dependent Clp protease ATP-binding subunit ClpX
24	Proteasome_bacterial----ATP-dependent Clp protease proteolytic subunit (EC 3.4.21.92)
25	Proteasome_bacterial----ATP-dependent hsl protease ATP-binding subunit HslU
26	Proteasome_bacterial----ATP-dependent protease HslV (EC 3.4.25.-)
**27**	Proteasome_bacterial----ATP-dependent protease La (EC 3.4.21.53) Type I
28	Proteasome_bacterial----ATP-dependent protease La (EC 3.4.21.53) Type II
29	Proteolysis_in_bacteria,_ATP-dependent----ATP-dependent Clp protease adaptor protein ClpS
30	Proteolysis_in_bacteria,_ATP-dependent----ATP-dependent Clp protease ATP-binding subunit ClpA
31	Proteolysis_in_bacteria,_ATP-dependent----ATP-dependent Clp protease, ATP-binding subunit ClpC
32	Proteolysis_in_bacteria,_ATP-dependent----ATP-dependent Clp protease ATP-binding subunit ClpX
33	Proteolysis_in_bacteria,_ATP-dependent----ATP-dependent Clp protease proteolytic subunit (EC 3.4.21.92)
34	Proteolysis_in_bacteria,_ATP-dependent----ATP-dependent hsl protease ATP-binding subunit HslU
35	Proteolysis_in_bacteria,_ATP-dependent----ATP-dependent protease HslV (EC 3.4.25.-)
**36**	Proteolysis_in_bacteria,_ATP-dependent----ATP-dependent protease La (EC 3.4.21.53)
**37**	Proteolysis_in_bacteria,_ATP-dependent----ATP-dependent protease La (EC 3.4.21.53) Type I
38	Proteolysis_in_bacteria,_ATP-dependent----ATP-dependent protease La (EC 3.4.21.53) Type II
39	Aminopeptidases_(EC_3.4.11.-)----Cytosol aminopeptidase PepA (EC 3.4.11.1)
40	Aminopeptidases_(EC_3.4.11.-)----Membrane alanine aminopeptidase N (EC 3.4.11.2)
41	Aminopeptidases_(EC_3.4.11.-)----Xaa-Pro aminopeptidase (EC 3.4.11.9)
42	Metallocarboxypeptidases_(EC_3.4.17.-)----D-alanyl-D-alanine carboxypeptidase (EC 3.4.16.4)
43	Metallocarboxypeptidases_(EC_3.4.17.-)----Thermostable carboxypeptidase 1 (EC 3.4.17.19)
44	Protein_degradation----Aminopeptidase YpdF (MP-, MA-, MS-, AP-, NP- specific)
45	Protein_degradation----Dipeptidyl carboxypeptidase Dcp (EC 3.4.15.5)
46	Protein_degradation----Oligopeptidase A (EC 3.4.24.70)
47	Serine_endopeptidase_(EC_3.4.21.-)----Prolyl endopeptidase (EC 3.4.21.26)

No. refers to protein-coding gene number as shown in Fig. 6. Protein-coding genes with the highest abundances are underlined and in bold

### Nitrification

**Fig. 7:**
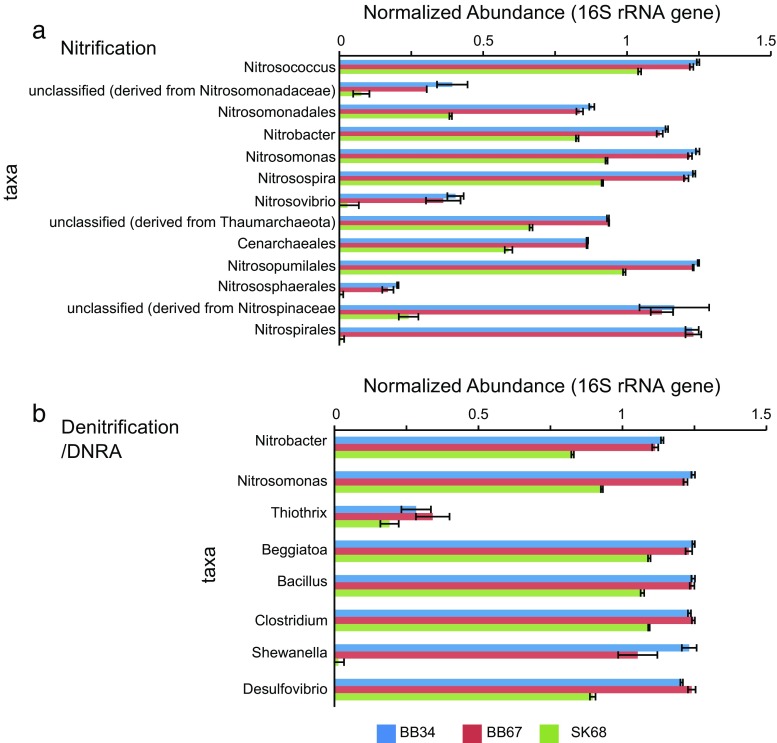
Normalized 16S rRNA gene abundances of microorganisms detected in SK and BB samples. *Bar charts* showing normalized 16S rRNA gene abundances of microorganisms able to perform **a** nitrification, **b** denitrification and DNRA. BB34 refers to sample 3–4 cmbsf (centimetre below sea-floor), BB67 refers to sample 6–7 cmbsf and SK68 refers to sample 6–8 cmbsf). Values for samples were scaled from 0 (minimum value) to 1 (maximum value) using a uniform scaling method implemented in the MG-RAST pipeline. Forward and reverse scaled abundances were averaged and a single value is reported per sample along with its standard deviation. The *bar charts* were generated using the MD5NR database, a maximum *e* value cut-off of 1e-05, minimum identity cut-off of 60% and a minimum alignment length cut-off 15

The 16S rRNA genes showed that unclassified *Thaumarchaeota* (AOA), *Nitrosospherales* (AOA), *Nitrosopumilales* (AOA), *Nitrosomonas* (AOB/NOB), *Nitrosospira* (AOB/NOB), *Nitrosovibrio* (AOB/NOB), *Nitrosococcus* (AOB/NOB), *Nitrospira* (NOB), *Nitrobacter* (NOB) and *Nitrospina* (NOB) were present in both samples (Fig. 7a). Comparison of AOA and AOB 16S rRNA gene abundances showed AOB and AOA abundances were not significantly different in BB and SK sediments (unpaired *t* test; *P* > 0.05).

The genes *amo* and the *hao* genes were present in all samples (≤0.02 normalized abundances). AMO and HAO are involved in the first step of nitrification in AOB; however, no *hao* homologue has been found in archaeal genomes (Stahl and de la Torre 2012). We also include additional supporting pyrosequencing results that show evidence for the high abundance of *Thaumarchaeota* in BB sediments relative to other archaea (Fig. S1).

### Dissimilatory NO_3_^−^ Reduction to NH_4_^+^ (DNRA)

**Fig. 8:**
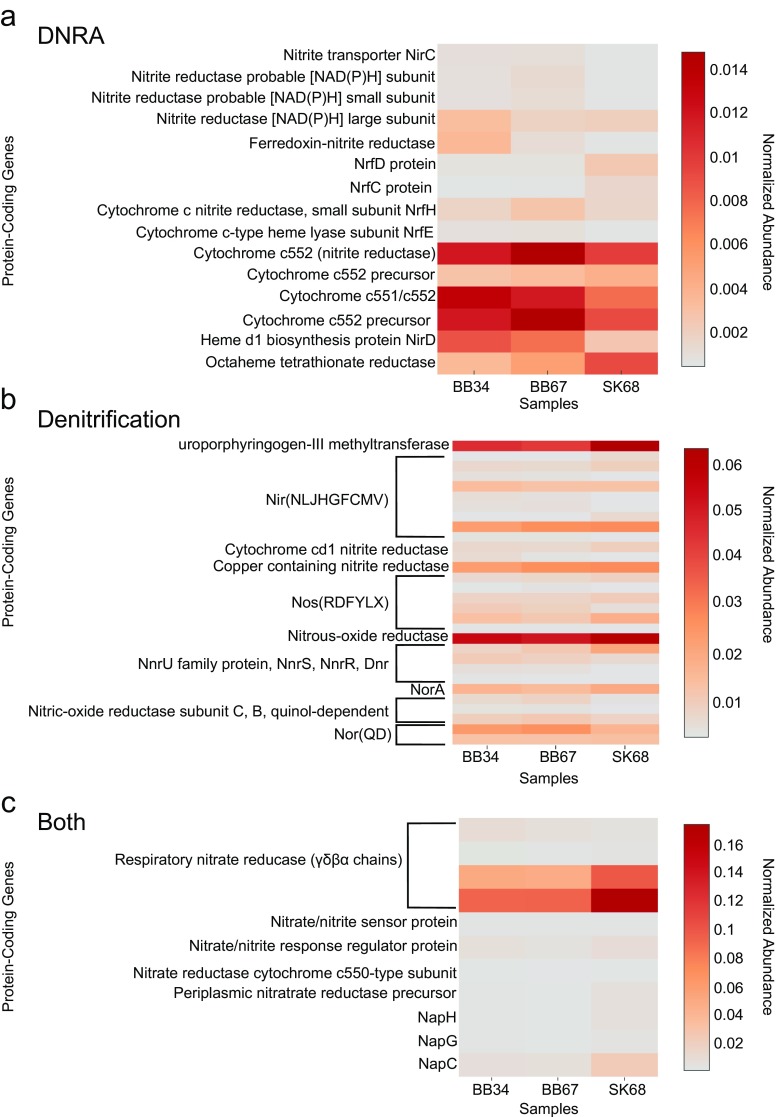
Heat maps showing normalized putative protein-coding gene abundances predicted to be involved in **a** DNRA, **b** denitrification or **c** both, detected in SK and BB metagenomes. BB34 refers to sample 3–4 cmbsf (centimetre below sea-floor), BB67 refers to sample 6–7 cmbsf and SK68 refers to sample 6–8 cmbsf. Values for samples were scaled from 0 (minimum value) to 1 (maximum value) using a uniform scaling method implemented in the MG-RAST pipeline. Forward and reverse abundances were averaged and a single value is reported per sample

The abundance of DNRA protein-coding genes was similar within BB samples (unpaired *t* test; *P* > 0.05) and between SK and BB samples (unpaired *t* test; *P* > 0.05) (Fig. 8a). In *Proteobacteria* including *Shewanella oneidensis* MR-1, NrfA (nitrite reductase) can associate with different types of Nrf proteins as part of the DNRA pathway (Baggs and Phillipot 2011; Cruz-García et al. 2007). Similarly, in δ and ε-*Proteobacteria*, NrfH can donate electrons to NrfA while in γ-*Proteobacteria*, NrfC and D are alternative electron donors (Baggs and Philippot 2011). *nrfA* (cytochrome c552 NO_2_^−^ reductase) was present at high abundance in all samples (Fig. 8a). Accessory protein-coding *nrfC*, *D*, *H* and *E* genes were present in low abundance (Fig. 8a). In *Shewanella oneidensis* MR-1, an octaheme tetrathionate reductase (Otr) (Atkinson et al. 2007) is active for DNRA. Protein-coding *otr* genes were also observed in all samples (Fig. 8a).

**Fig. 9:**
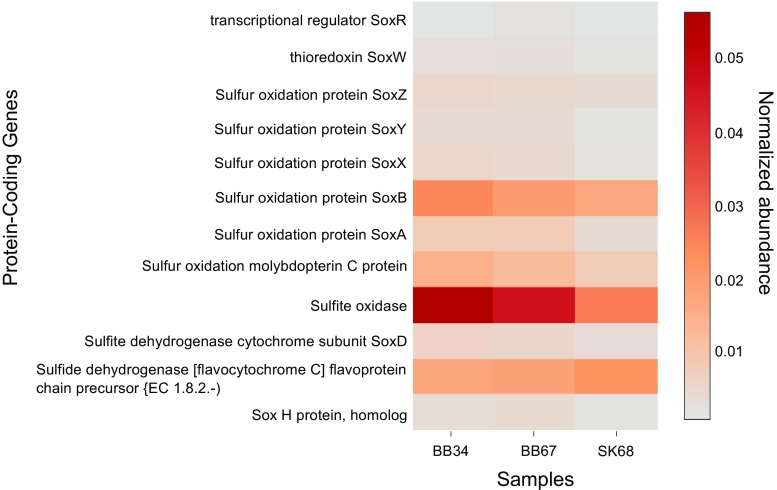
Heat map showing normalized protein-coding gene abundances predicted to be involved in sulphur oxidation metabolism detected in SK and BB metagenomes. BB34 refers to sample 3–4 cmbsf (centimetre below sea-floor), BB67 refers to sample 6–7 cmbsf and SK68 refers to sample 6–8 cmbsf. Values for samples were scaled from 0 (minimum value) to 1 (maximum value) using a uniform scaling method implemented in the MG-RAST pipeline. Forward and reverse abundances were averaged and a single value is reported per sample

Dissimilatory NO_3_^−^ reduction can also take place chemolithoautotrophically, e.g. some *Beggiatoa* are capable of DNRA, storing NO_3_^−^ in vacuoles and coupling its reduction to NH_4_^+^, to sulphide oxidation (Muβmann et al. 2003; Preisler et al. 2007 and references therein; Schulz-Vogt 2011). 16S rRNA *Beggiatoa* gene relative abundances appeared slightly higher in BB vs. SK samples (Fig. 7b). Sulphide oxidation (*soxADZBX*) genes were also detected in all samples (Fig. 9).

**Fig. 10:**
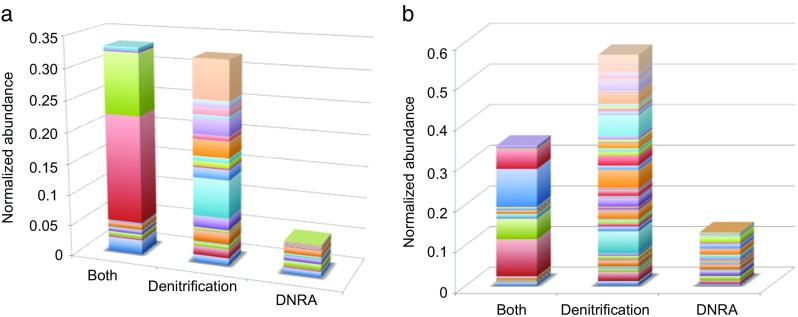
3-D charts showing the normalized abundances of genes predicted to be involved in denitrification and DNRA pathways (referred to as both), only in the denitrification pathway and only in the DNRA pathway in **a** SK and **b** BB samples

Fermentative bacteria such as *Clostridium* and *Bacillus* spp*.* can use NO_2_^−^ as an electron acceptor during fermentative growth, employing the use of cytoplasmic (NADH) nitrite reductase NirBD proteins (Cabello et al. 2009), thereby contributing to production of NH_4_^+^ (Baggs and Phillipot 2011). 16S rRNA abundances showed they were more abundant in BB samples (Fig. 7b). Protein-coding genes for NirB (nitrite reductase [NAD(P)H] large subunit) and NirD were also present in all samples (Fig. 8a). Comparison of DNRA protein-coding gene abundances (≤0.014 normalized abundance) (Fig. 8a) to denitrification protein-coding gene abundances (≥0.01 normalized abundance) (Fig. 8b) within samples showed genes for DNRA were significantly less abundant (unpaired *t* test; *P* < 0.05) (Fig. 10a, b).

### Denitrification Pathway

16S rRNA genes of *Thiothrix* were present in all samples (Fig. 7b). At both sites, protein-coding genes for denitrification were found. In BB samples, the abundances of protein-coding genes involved in denitrification were similar between the two depths (unpaired *t* test; *P* > 0.05) (Fig. 8b). Similar abundances of protein-coding genes were also observed between SK and BB samples (unpaired *t* test; *P* > 0.05). Genes including *nir*K (copper-containing nitrite reductase), *nirS* (cytochrome cd1 nitrite reductase) (Helen et al. 2016) and *nirV* and *nirN* genes were detected in all samples (Fig. 8b). While most α, β and γ-*Proteobacteria* use NirK to reduce NO_2_^−^ to NO, other bacteria use NirS as an alternative enzyme and NirN is a homologue of NirS (van Spanning 2011). Additionally, NirV is sometimes associated with NirK, and may incorporate copper into the redox centre of NirK (van Spanning 2011). Nor genes (*norQD*) were present (Fig. 8b) along with nitric oxide reductase (NOR) subunit C and B genes (EC 1.7.99.7). The catalytic NOR enzyme encoded by *norCB* can be co-expressed with accessory genes *norQ*, *norD* (although their function remains elusive). Additionally, *nos* genes (*nosRDFYLX*) and a nitrous oxide reductase gene (EC 1.7.99.6) were present in all samples (Fig. 8b). NosFY and D are ABC transporters that are usually linked to expression of NosZ, the main multi-copper enzyme involved in N_2_O reduction, and NosR is required for transcription of NosZ (Spiro 2012). Additionally, NosL, a membrane anchored copper protein, and NosX, a periplasmic flavoprotein, may serve as accessory proteins to NosZ (Spiro 2012). We found that with the exception of *nirK*, none of the other genes that were abundant in our DNRA or denitrification heat maps are known to occur in multiple copies. When the abundance of this gene was left out of the analysis, the results of the heat map remained the same.

### Genes Involved in Denitrification and DNRA Pathways

Genes in the Nar and Nap families that are involved in the first step of the denitrification and DNRA pathways were detected in all samples but were not significantly different between samples (unpaired *t* test; *P* > 0.05) (Fig. 8c). The *narGH* genes, which encode the alpha and beta subunit of the Nar enzyme, were the most abundant genes detected out of all denitrification and DNRA protein-coding genes (Fig. 8c). The *narI* gene (gamma subunit), the chaperone encoding *narJ* (delta subunit) gene, nitrate response regulator protein (*narQ*/*P*) and nitrate/nitrite sensor protein (*narX*/*L*) were also detected but at lower abundances (Fig. 8c) (Blasco et al. 1998; Cabello et al. 2009). Among the Nap family of genes, a *napC* (a membrane tetraheme c-type cytochrome), *napA* (NO_3_^−^ reductase) and accessory proteins *napG* and *H* were detected. The NapC protein is an essential component of certain δ and γ-*Proteobacteria* (including *Desulfovibrio* spp. and MR-1) and donates electrons to NapA (Potter and Cole 1999; Brondijk et al. 2002; Simon 2002; Chen and Wang 2015). However, an alternative pathway in these interactions can also include NapG and H (Baggs and Phillipot 2011).

## Discussion

### Geochemistry

Modelling results suggest ammonification and nitrification could be active processes in the BB (3–4 cm) sample. While ammonification occurs most likely throughout the SK core due to organic matter degradation, it is masked by even higher rates of nitrification within the top 5 cm (Fig. 5b). A double peak in NH_4_^+^ consumption may result from non-steady-state conditions, most likely due to bioturbation. In addition, modelling results indicate denitrification and DNRA could take place in the SK (8–10 cm) and BB (6–7 cm) samples (Fig. 5a, c). Sediments with a high organic carbon input and nitrogen limitation are predicted to favour DNRA over denitrification based on previous studies (Giblin et al. 2013; Algar and Vallino 2014). Both the SK and BB receive high inputs of organic matter (sedimentation rate ~4 mm year^−1^ SK; ~1.1–1.6 mm year^−1^ BB; van Weering et al. 1987; Mattila et al. 2006). However, the quality of organic matter could also be an important factor. In the SK and BB, total organic carbon (TOC) is available throughout both sediments (Reyes et al. 2016) and could stimulate heterotrophic activity. However, a more refractory organic matter in the BB could also slow down heterotrophic activity. Total nitrogen (TN) is also readily available in both sediments (Reyes et al. 2016) resulting in a C/N ratio of ~10 and 30 for SK and BB, respectively. Due to the high input of organic matter and nitrogen in both sites, denitrification could possibly dominate over DNRA.

### Metagenome

When interpreting results derived from DNA in sediments, it is important to keep in mind certain physical processes that could influence the distribution of genes. One process is sedimentation, which occurs as new sediment layers are continuously deposited over time, thereby shifting the biogeochemical zones. Another process that could shift biogeochemical zones is storm events. During storms, sediments from the surface could be resuspended in the water column, bringing deeper zones to the surface. Thus, extra-cellular DNA or cellular DNA could end up preserved in a different biogeochemical layer. Another important sedimentary process to be aware of is bioturbation. Sedimentary layers could become homogenized by bioturbation, thereby influencing the distribution (and hence abundance) of genes in sediments (Laverock et al. 2014). Although the metagenome data do not directly indicate activity with respect to a particular organism or pathway, the presence of protein-coding genes in the sediment represents a metabolic potential. In this case, the presence of these genes represents a metabolic potential with respect to nitrogen metabolism.

Since many dissimilatory bacteria are involved in amino acid and protein degradation thereby contributing to ammonification, perhaps this explains the higher abundance of ammonification genes over other types of nitrogen metabolism genes in both samples. In sediments of Landsort Deep (Baltic Sea), where DNRA and denitrification has been shown to be important, metagenome results also show that ammonification-coding genes are more abundant compared to genes involved in other nitrogen pathways (Thureborn et al. 2013). Moreover, bacterial proteases and hydratases could potentially contribute most to the release of NH_4_^+^ from proteins and amino acids during ammonification in samples from both sites (Table 1).

In other marine sediment studies, the abundance of AOA and AOB varies and the factors that determine whether one is more abundant than the other are not clear and may depend on many variables including salinity (Caffrey et al. 2007; Mosier and Francis 2008; Santoro et al. 2008), NH_4_^+^ availability (Smith et al. 2014) and spatial and temporal variations (Beman et al. 2012; Smith et al. 2015). From the 16S rRNA metagenomic results, it appears that mostly *Thaumarchaeota* and AOB could contribute to nitrification at both sites (Fig. 7a). *Thaumarchaeota* have been found to be abundant (Thureborn et al. 2013) and to play an important role in ammonia oxidation at other locations in the Baltic (Labrenz et al. 2010; Feike et al. 2012). Furthermore, DNA pyrosequencing results of these samples indicate *Thaumarchaeota*/*Nitrosopumilus* are abundant relative to other archaea and are the major archaeal type in BB sediments (Fig. S1). While a diversity of archaea is present in SK sediments, the BB appears to be dominated by *Thaumarchaeota* (Reyes and Noriega-Ortega 2016). Based on metagenomic comparisons between AOA and AOB, however, it remains unclear whether AOA or AOB are more important with respect to NH_3_-oxidation in BB and SK sediments.

In terms of denitrification, *Thiothrix* could have access to both thiosulphates and NO_3_^−^ in SK and BB sediments, allowing it to carry out thiosulphate oxidation coupled to NO_3_^−^ reduction (Meyer et al. 2007). Porewater results of BB samples showed a decrease in sulphate concentration, which overlapped with the zone of NO_3_^−^ reduction but no sulphide was present (Reyes et al. 2016). *Thiothrix* (including the isolate known to carry out this chemolithoautotrophic reaction) can live in freshwater and marine habitats (Trubitsyn et al. 2013), possibly explaining the presence of *Thiothrix* 16S rRNA genes in SK and BB samples. The presence of protein-coding genes related to sulphur oxidation and denitrification in both samples supports the idea that one way in which denitrification could occur in BB (6–7 cm) and SK (8–10 cm) samples is via a chemolithoautotrophic pathway.

Other organisms such as *Nitrosomonas* and *Nitrobacter*, which can carry out only certain steps of the denitrification process, could potentially be involved in reduction of NO_3_^−^ to NO_2_^−^ in BB (6–7 cm) and SK (8–10 cm) using *nar* genes. *Nitrosomonas* could also contribute to NO_3_^−^ reduction to N_2_ using *nap*, *nir* and *nor* genes in these same samples (Fig. 4).

Some marine and freshwater *Beggiatoa* have the ability to denitrify (Sweerts et al. 1990; Muβmann et al. 2007), and DNRA is well documented in *Beggiatoa* (Preisler et al. 2007 and references therein). *Beggiatoa* have been shown to be capable of surviving independently from external sources of sulphur and NO_3_^−^ for up to 2 weeks in laboratory experiments (Preisler et al. 2007). Protein-coding genes detected in all samples such as *otr* (Fig. 8b; MacGregor et al. 2013) and sulphide oxidation (*soxADZBX*) genes (Fig. 9) support the idea that chemolithoautotrophic microorganisms like *Beggiatoa* could be involved in DNRA in these sediments. Due to their metabolic versatility, it is less clear if *Shewanella* and *Desulfovibrio* could be involved in denitrification.

For similar reasons, it is unclear if *Clostridium* and *Bacillus* could be involved in DNRA in these sediments. Although we could not infer whether the above taxa could potentially be involved in fermentative DNRA, the presence of small and large NADH nitrite reductase subunit genes (Fig. 8b) supports the idea that DNRA could also occur via heterotrophic fermentation in these sediments.

When genes for denitrification, DNRA or genes involved in both processes were compared, genes involved in denitrification were significantly greater in abundance than those involved in DNRA (Fig. 10a, b). A study by Trimmer et al. (2013) found rates of denitrification to be high at Skagerrak sites (S4, S6, S8 and S9) neighbouring our site (Fig. 2) and the potential for DNRA to be negligible to moderate. Based on the presence of specific taxa, protein-coding genes and geochemistry results, denitrification could potentially be a more important pathway in the suboxic zone at both sites.

## Conclusions

In this study, we are able to provide a model for ammonification, nitrification, NO_3_^−^ reduction and denitrification processes in the SK and BB sediments based on the presence of corresponding genes (Fig. 4). Proteases and hydratases appeared to make up the bulk of ammonification genes at both sites. Genes associated with aerobic ammonia oxidation (*amo* and *hao*) were present and suggest AOA/AOB contribute to aerobic ammonia oxidation at the sediment-water interface at SK and BB. However, it remains unclear which one may have a more important role in both sediments. In addition, the presence of *nrfA*, *nirBD* and *otr* and NADH nitrite-reductase genes implies that DNRA could contribute to NH_4_^+^ production where NO_3_^−^ is available either via a respiratory or fermentative pathway. Genes for sulphide oxidation (s*oxADZBX*) could allow for *Thiothrix* and *Beggiatoa* to carry out chemolithoautotrophic NO_3_^−^ reduction coupled to thiosulphate or sulphide oxidation either via denitrification or DNRA. *Nitrosomonas* and *Nitrobacter* could contribute to NO_3_^−^ reduction using *nar, nap*, *nir* and *nor* genes near the surface where NO_3_^−^ is consumed. Biogeochemical and metagenomic results suggest denitrification could play the more important role in both sediments. Overall, these results show that protein-coding genes for these cycles are potentially operative in suboxic marine sediments at these sites. Our study offers the first in-depth metagenomic characterization of nitrogen cycling and associated genes of suboxic SK and BB sediments.

## Electronic supplementary material

ESM 1 (DOCX 15 kb)

Table S1 (DOCX 16 kb)

Table S2 (DOCX 15 kb)

Fig. S1 (EPS 336 kb)

## References

[CR1] Algar CK, Vallino JJ (2014). Predicting microbial nitrate reduction pathways in coastal sediments. Aquat Microb Ecol.

[CR2] Atkinson SJ, Moat CG, Reid GA, Chapman SK (2007). An octaheme c-type cytochrome from *Shewanella oneidensis* can reduce nitrate and hydroxylamine. FEBS Lett.

[CR3] Baggs E, Phillipot L (2011) Nitrous oxide production in the terrestrial environment. In: Moir JWB (ed) Nitrogen cycling in bacteria: molecular analysis. Caister Academic Press, Norfolk

[CR4] Bale NJ, Villanueva L, Hopmans EC, Schouten S, Sinninghe Damsté JS (2013). Different seasonality of pelagic and benthic Thaumarchaeota in the North Sea. Biogeosciences.

[CR5] Beman JM, Bertics VJ, Braunschweiler T, Wilson JM (2012). Quantification of ammonia oxidation rates and the distribution of ammonia-oxidizing *Archaea* and *Bacteria* in marine sediment depth profiles from Catalina Island, California. Front Microbiol.

[CR6] Berg P, Risgaard-Petersen N, Rysgaard S (1998). Interpretation of measured concentration profiles in sediment pore water. Limnol Oceanogr.

[CR7] Blasco F, Dos Santos JP, Magalon A, Frixon C, Guigliarelli B, Santini CL, Giordano G (1998). NarJ is a specific chaperone required for molybdenum cofactor assembly in nitrate reductase A of *Escherichia coli*. Mol Microbiol.

[CR8] Bolger AM, Lohse M, Usadel B (2014) Trimmomatic: a flexible trimmer for Illumina sequence data. Bioinformatics 30:2114–2120 10.1093/bioinformatics/btu170 PMC4103590 24695404

[CR9] Boudreau BP (1997). Diagenetic models and their implementation.

[CR10] Braithwaite DT, Keegan KP (2013) matR: metagenomics analysis tools for R. R package version 0.9.9

[CR11] Braker G, Zhou J, Wu L, Devol AT, Tiedje JM (2000). Nitrite reductase genes (*nirK* and *nirS*) as functional markers to investigate diversity of denitrifying bacteria in pacific northwest marine sediment communities. Appl Environ Microbiol.

[CR12] Brondijk THC, Fiegen D, Richardson DJ, Cole JA (2002). Roles of NapF, NapG and NapH subunits of the *Escherichia coli* periplasmic nitrate reductase in ubiquinol oxidation. Mol Microbiol.

[CR13] Cabello P, Roldán MD, Castillo F, Moreno-Vivián C (2009) Nitrogen cycle. In: Schaechter M (ed) Encyclopaedia of microbiology, 3rd edn. Academic Press, London

[CR14] Caffrey JM, Bano N, Kalanetra K, Hollibaugh JT (2007). Ammonia oxidation and ammonia-oxidizing bacteria and archaea from estuaries with differing histories of hypoxia. ISME J.

[CR15] Canfield DE, Thamdrup B, Hansen JW (1993). The anaerobic degradation of organic matter in Danish coastal sediments: iron reduction, manganese reduction, and sulfate reduction. Geochim Cosmochim Acta.

[CR16] Cantera JJL, Stein LY (2007). Molecular diversity of nitrite reductase genes (*nirK*) in nitrifying bacteria. Environ Microbiol.

[CR17] Chen Y, Wang F (2015). Insights on nitrate respiration by *Shewanella*. Front Mar Sci.

[CR18] Cruz-García C, Murray AE, Klappenbach JA, Stewart V, Tiedje JM (2007). Respiratory nitrate ammonification by *Shewanella oneidensis* MR-1. J Bacteriol.

[CR19] Dang H, Zhang X, Sun J, Li T, Zhang Z, Yang G (2008). Diversity and spatial distribution of sediment ammonia-oxidizing crenarchaeota in response to estuarine and environmental gradients in the Changjiang Estuary and East China Sea. Microbiology.

[CR20] Delwiche CC (1970). The nitrogen cycle. Sci Am.

[CR21] Dini Anderote F, Jiménez DJ, Chaves D, Franco Dias AC, Luvizotto DM, Dini-Anderote F, Fasanella CC, Lopez MV, Baena S, Taketani RG, de Melo IS (2012). The microbiome of Brazilian mangrove sediments as revealed by metagenomics. PLoS One.

[CR22] Feike J, Jürgens K, Hollibaugh JT, Krüger S, Jost G, Labrenz M (2012). Measuring unbiased metatranscriptomics in suboxic waters of the central Baltic Sea using a new in situ fixation system. ISME J.

[CR23] Francis CA, Beman JM, Kuypers MMM (2007). New processes and players in the nitrogen cycle: the microbial ecology of anaerobic and archaeal ammonia oxidation. ISME J.

[CR24] Francis CA, Roberts KJ, Beman JM, Santoro A, Oakley BB (2005). Ubiquity and diversity of ammonia-oxidizing archaea in water columns and sediments of the ocean. Proc Natl Acad Sci U S A.

[CR25] Freitag TE, Chang L, Prosser JI (2006). Changes in the community structure and activity of betaproteobacterial ammonia-oxidizing sediment bacteria along a freshwater–marine gradient. Environ Microbiol.

[CR26] Froelich PN, Klinkhammer GP, Bender ML, Luedtke NA, Heath GR, Cullen D, Dauphin P (1979). Early diagenesis of organic matter in pelagic sediments of the eastern equatorial Atlantic: suboxic diagenesis. Geochim Cosmochim Acta.

[CR27] Flemming BW, Delafontaine MT (2000). Mass physical properties of muddy intertidal sediments: some applications, misapplications and non-applications. Cont Shelf Res.

[CR28] Giblin AE, Weston NB, Banta GT, Tucker J, Hopkinson CS (2010). The effects of salinity on nitrogen losses from an oligohaline estuarine sediment. Estuar Coasts.

[CR29] Giblin AE, Tobias CR, Song B, Weston N, Banta GT, Rivera-Monroy VH (2013). The importance of dissimilatory nitrate reduction to ammonium (DNRA) in the nitrogen cycle of coastal ecosystems. Oceanography.

[CR30] Grasshoff K, Kremling K, Ehrhardt M (1999) Determination of nitrate. Determination of ammonia. In: Grasshoff K, Kremling K, Ehrhardt M et al (eds) Methods of seawater analysis. Wiley-VCH Verlag GmbH, Weinheim

[CR31] Herbert RA (1999). Nitrogen cycling in coastal marine ecosystems. FEMS Microbiol Rev.

[CR32] Helen D, Kim H, Tytgat B, Anne W (2016). Highly diverse nirK genes comprise two major clades that harbor ammonium producing denitrifiers. BMC Genomics.

[CR33] Iversen N, Jørgensen BB (1993). Diffusion coefficients of sulfate and methane in marine sediments: influence of porosity. Geochim Cosmochim Acta.

[CR34] Jørgensen KS (1989). Annual pattern of denitrification and nitrate ammonification in estuarine sediment. Appl Environ Microbiol.

[CR35] Keegan KP (2015) matR-apps. GitHub repository. http://github.com/DrOppenheimer/matR-apps. Accessed 7 April 2015

[CR36] Kimes NE, Callaghan AV, Aktas DF, Smith WL, Sunner J, Golding BT, Drozdowska M, Hazen TC, Suflita JM, Morris PJ (2013). Metagenomic analysis and metabolite profiling of deep-sea sediments from the Gulf of Mexico following the Deepwater Horizon oil spill. Front Microbiol.

[CR37] Könneke M, Bernhard AE, de la Torre JR, Walker CB, Waterbury JB, Stahl DA (2005). Isolation of an autotrophic ammonia-oxidizing marine archaeon. Nature.

[CR38] Labrenz M, Sintes E, Toetzke F, Zumsteg A, Herndl GJ, Seidler M, Jürgens K (2010). Relevance of a crenarchaeotal subcluster related to Candidatus *Nitrosopumilus maritimus* to ammonia oxidation in the suboxic zone of the central Baltic Sea. ISME J.

[CR39] Laverock B, Tait K, Gilbert JA, Osborn AM, Widdicombe S (2014). Impacts of bioturbation on temporal variation in bacterial and archaeal nitrogen-cycling gene abundance in coastal sediments. Environ Microbiol.

[CR40] Lettmann KA, Riedinger N, Ramlau R, Knab N, Böttcher ME, Khalili A, Wolff JO, Jørgensen BB (2012). Estimation of biogeochemical rates from concentration profiles: a novel inverse method. Estuar Coast Shelf Sci.

[CR41] Lueders T, Manefield M, Friedrich MW (2004). Enhanced sensitivity of DNA- and rRNA-based stable isotope probing by fractionation and quantitative analysis of isopycnic centrifugation gradients. Environ Microbiol.

[CR42] Lücker S, Wagner M, Maixner F, Pelletier E, Koch H, Vacherie B, Rattei T, Damsté JS, Spieck E, Le Paslier D, Daims H (2010). A *Nitrospira* metagenome illuminates the physiology and evolution of globally important nitrite-oxidizing bacteria. Proc Natl Acad Sci U S A.

[CR43] Mattila J, Kankaapää H, Ilus E (2006) Estimation of recent sediment accumulation rates in the Baltic Sea using artificial radionuclides 137Cs and 239,240Pu as time markers. Boreal Environ Res 11:96–107

[CR44] Meyer B, Imhoff J, Kuever J (2007). Molecular analysis of the distribution and phylogeny of the *soxB* gene among sulfur-oxidizing bacteria-evolution of the Sox sulfur oxidation enzyme system. Environ Microbiol.

[CR45] Meyer F, Paarmann D, D’Souza M, Olson R, Glass EM, Kubal M, Paczian T, Rodriguez A, Stevens R, Wilke A, Wilkening J, Edwards RA (2008). The metagenomics RAST server-a public resource for the automatic phylogenetic and functional analysis of metagenomes. BMC Bioinformatics.

[CR46] MacGregor BJ, Biddle JF, Harbort C, Matthysse AG, Teske A (2013). Sulfide oxidation, nitrate respiration, carbon acquisition, and electron transport pathways suggested by the draft genome of a single orange Guaymas Basin *Beggiatoa* (Cand. Maribeggiatoa) sp. filament. Mar Genomics.

[CR47] Middelburg JJ, Vlug T, van der Nat JWAF (1993). Organic matter mineralization in marine systems. Glob Planet Chang.

[CR48] Michotey V, Méjean V, Bonin P (2000). Comparison of methods for quantification of cytochrome cd1-denitrifying bacteria in environmental marine samples. Appl Environ Microbiol.

[CR49] Mosier AC, Francis CA (2008). Relative abundance and diversity of ammonia-oxidizing archaea and bacteria in the San Francisco Bay estuary. Environ Microbiol.

[CR50] Muβmann M, Shultz HN, Strotmann B, Kjaer T, Nielsen LP, Roselló-Mora RA, Amann RI, Jørgensen BB (2003). Phylogeny and distribution of nitrate-storing *Beggiatoa* spp. in coastal marine sediments. Environ Microbiol.

[CR51] Muβmann M, Hu FZ, Richter M, de Beer D, Preisler A, Jørgensen BB, Huntemann M, Glӧckner FO, Amann R, Koopman WJH, Lasken RS, Janto B, Hogg J, Stoodley P, Boissy R, Ehrlich GD (2007). Insights into the genome of large sulfur bacteria revealed by analysis of single filaments. PLoS Biol.

[CR52] Norton JM, Alzerreca JJ, Suwa Y, Klotz MG (2001). Diversity of ammonia monooxygenase operon in autotrophic ammonia-oxidizing bacteria. Arch Microbiol.

[CR53] Pauleta SR, Dell'acqua S, Moura I (2013). Nitrous oxide reductase. Coord Chem Rev.

[CR54] Potter LC, Cole JA (1999). Essential roles for the products of the *napABCD* genes, but not nap FGH in periplasmic nitrate reduction by *Escherichia coli* K-12. Biochem J.

[CR55] Pitcher A, Wuchter C, Siedenberg K, Schouten S, Sinninghe Damsté JS (2011). Crenarchaeol tracks winter blooms of ammonia-oxidizing Thaumarchaeota in the coastal North Sea. Limnol Oceanogr.

[CR56] Preisler A, de Beer D, Lichtschlag A, Lavik G, Boetius A, Jørgensen BB (2007). Biological and chemical sulfide oxidation in a *Beggiatoa* inhabited marine sediment. ISME J.

[CR57] R Core Team (2013) A language and environment for statistical computing. R Foundation for Statistical Computing, Vienna. https://www.r-project.org/. Accessed 7 April 2015

[CR58] Reyes C, Noriega-Ortega B (2016) Report: microbial diversity of Baltic Sea and North Sea sediments based on pyrosequencing results. FigShare. doi:10.6084/m9.figshare.3171442.v1

[CR59] Reyes C, Dellwig O, Dähnke K, Gehre M, Noriega-Ortega BE, Böttcher ME, Meister P, Friedrich MW (2016). Bacterial communities potentially involved in iron-cycling in Baltic Sea and North Sea sediments revealed by pyrosequencing. FEMS Microbiol Ecol.

[CR60] Santoro AE, Francis CA, De Sieyes NR, Boehm AB (2008). Shifts in the relative abundance of ammonia-oxidizing bacteria and archaea across physicochemical gradients in a subterranean estuary. Environ Microbiol.

[CR61] Seeberg-Elverfeldt J, Schlüter M, Feseker T, Kӧlling M (2005). Rhizon sampling of porewaters near the sediment-water interface of aquatic systems. Limnol Oceanogr Methods.

[CR62] Scala DJ, Kerkhof LJ (1998). Nitrous oxide reductase (*nosZ*) gene-specific PCR primers for detection of denitrifiers and three *nosZ* genes from marine sediments. FEMS Microbiol Lett.

[CR63] Scala DJ, Kerkhof LJ (1999). Diversity of nitrous oxide reductase (*nosZ*) genes in continental shelf sediments. Appl Environ Microbiol.

[CR64] Schleper C, Nicol GW (2010) Diversity, distribution and activity of ammonia-oxidizing archaea in the environment: AOA in sediments. In: Poole RK (ed) Advances in microbial physiology, vol 57. Academic Press, London

[CR65] Schulz HD, Zabel M (2006) Quantification of early diagenesis: dissolved constituents in pore water and signals in the solid phase. In: Shulz HD, Zabel M (eds) Marine geochemistry. Springer-Verlag, Berlin Heidelberg

[CR66] Schulz-Vogt HN (2011) Beggiatoa. In: Reitner J, Thiel V (eds) Encyclopedia of geobiology. Springer, Netherlands

[CR67] Scott NM, Hess M, Bouskill NJ, Mason OU, Jansson JK, Gilbert JA (2014). The microbial nitrogen cycling potential is impacted by polyaromatic hydrocarbon pollution of marine sediments. Front Microbiol.

[CR68] Shapleigh JP (2013) Denitrifying prokaryotes. In: Rosenberg E, DeLong EF, Lory S, Stackebrandt E, Thompson F (eds) The prokaryotes: prokaryotic physiology and biochemistry. Springer-Verlag, Berlin Heidelberg

[CR69] Simon J (2002). Enzymology and bioenergetics of respiratory nitrite ammonification. FEMS Microbiol Rev.

[CR70] Smith JM, Casciotti KL, Chavez FP, Francis CA (2014). Differential contributions of archaeal ammonia oxidizer ecotypes to nitrification in coastal surface waters. ISME J.

[CR71] Smith JM, Mosier AC, Francis CA (2015). Spatiotemporal relationships between the abundance, distribution, and potential activities of ammonia-oxidizing and denitrifying microorganisms in intertidal sediments. Microb Ecol.

[CR72] Spieck E, Bock E (2005) Nitrifying bacteria. In: Brenner DJ, Krieg NR, Staley JT, Sc.D GMG (eds) Bergey’s manual of systematic bacteriology, vol 2. The *Proteobacteria* Part A Introductory Essays. Springer, USA

[CR73] Spiro S (2012). Nitrous oxide production and consumption: regulation of gene expression by gas-sensitive transcription factors. Philos Trans R Soc B.

[CR74] Söderlund R, Svensson BH (1976). The global nitrogen cycle. Ecol Bull.

[CR75] Soonmo A, Gardner WS (2002). Dissimilatory nitrate reduction to ammonium (DNRA) as a nitrogen link, versus denitrification as a sink in a shallow estuary (Laguna Madre/Baffin Bay, Texas). Mar Ecol Prog Ser.

[CR76] Stahl DA, de la Torre JR (2012). Physiology and diversity of ammonia-oxidizing archaea. Annu Rev Microbiol.

[CR77] StatPlus:mac. Microsoft Excel 2011. Analyst Soft Inc., California, USA

[CR78] Sweerts J, Debeer D, Nielsen LP, Verdouw H, Van Den Heuvel JC, Cohen Y, Cappenberg TE (1990). Denitrification by sulfur oxidizing *Beggiatoa* spp. mats on fresh-water sediments. Nature.

[CR79] Teske A, Alm E, Regan JM, Toze S, Rittmann BE, Stahl DA (1994). Evolutionary relationships among ammonia- and nitrite-oxidizing bacteria. J Bacteriol.

[CR80] Therkildsen MS, King GM, Lomstein BA (1996). Urea production and turnover following the addition of AMP, CMP, RNA and a protein mixture to a marine sediment. Aquat Microb Ecol.

[CR81] Therkildsen MS, Isaksen MF, Lomstein BA (1997). Urea production by the marine bacteria *Delaya venusta* and *Psuedomonas stutzeri* grown in minimal medium. Aquat Microb Ecol.

[CR82] Thureborn P, Lundin D, Plathan J, Poole AM, Sjörn BM, Sjölin S (2013). A metagenomics transect into the deepest part of the Baltic Sea reveals clear stratification of microbial functional capacities. PLoS One.

[CR83] Trimmer M, Engstrom P, Thamdrup B (2013). Stark contrast in denitrification and anammox across the deep Norwegian trench in the Skagerrak. Appl Environ Microbiol.

[CR84] Trubitsyn IV, Andreevskish ZG, Yurevich LI, Belousova EV, Tutukina MN, Merkel AY, Dubinina GA, Grabovich MY (2013). Capacity for nitrate respiration as a new aspect of metabolism of the filamentous sulphur bacteria of the genus *Thiothrix*. Microbiology.

[CR85] van Spanning RJM (2011) Structure, function, regulation and evolution of the nitrite and nitrous oxide reductases: denitrification enzymes with a beta-propeller fold. In: Moir JWB (ed) Nitrogen cycling bacteria: molecular analysis. Caister Academic Press, Norfolk

[CR86] van Weering TCE, Berger GW, Kalf J (1987). Recent sediment accumulation in the Skagerrak, Northeastern North Sea. Neth J Sea Res.

[CR87] Walker CB, de la Torre JR, Klotz MG, Urakawa H, Pinel N, Arp DJ, Brochier-Armanet C, Chain PS, Chan PP, Gollabgir A, Hemp J, Hügler M, Karr EA, Kӧnneke M, Shin M, Lawton TJ, Lowe T, Martens-Habena W, Sayavedra-Sota LA, Lang D, Sievert SM, Rosenzweig AC, Manning G, Stahl DA (2010). *Nitrosopumilus maritimus* genome reveals unique mechanisms for nitrification and autotrophy in globally distributed marine crenarchaea. Proc Natl Acad Sci U S A.

[CR88] Ward BB (2013) Nitrification. In: Reference module in earth systems and environmental sciences. Elsevier. doi:10.1016/B978-0-12-409548-9.00697-7

[CR89] Wilke A, Glass EM, Bischof J, Braithwaite D, Gerlach W, Harrison T, Keegan K, Paczian T, Trimble WL, Meyer F (2015) MG-RAST Manural for version 3.6, revision 3. ftp://ftp.metagenomics.anl.gov/data/manual/mg-rast-manual.pdf. Accessed 2 Oct 2015

[CR90] Winogradsky S (1890). Recherches sur les organismes de la nitrification. Ann Inst Pasteur.

[CR91] Wuchter C, Abbas B, Coolen MJL, Herfort L, Bleijswijk J, van Timmers P, Strous M, Teira E, Herndle GJ, Middelburg JJ, Schouten S, Damsté S (2006). Archaeal nitrification in the ocean. Proc Natl Acad Sci U S A.

[CR92] Xie W, Wang F, Guo L, Chen Z, Sievert SM, Meng J, Huang G, Li Y, Yan Q, Wu S, Wang X, Chen S, He G, Xiao X, Xu A (2011). Comparative metagenomics of microbial communities inhabiting deep-sea hydrothermal vent chimneys with contrasting chemistries. ISME J.

