# Supplementary material for: Nitrogen Metabolism Genes from Temperate Marine Sediments
Source: Mar Biotechnol (NY). 2017 Mar 10;19(2):175–90. doi: 10.1007/s10126-017-9741-0 (PMC5405112; doi:10.1007/s10126-017-9741-0)
Supplement: Supplementary file 1 — (DOCX 15 kb) [file 10126_2017_9741_MOESM1_ESM.docx]

**Supplementary Material**

**Nitrogen Metabolism Genes from Temperate Marine Sediments**

Carolina Reyes (1) *#, Dominik Schneider (2), Marko Lipka (3), Andrea Thürmer (2), Michael E. Böttcher (3), Michael W. Friedrich (1)

(1) University of Bremen, Microbial Ecophysiology, Leobener Strasse, D-28359 Bremen, Germany (2) University of Göttingen, Department of Genomic and Applied Microbiology, Grisebachstrasse 8, D-37077, Göttingen, Germany (3) Leibniz Institute for Baltic Sea Research (IOW), Geochemistry and Stable Isotope Biogeochemistry Group, Seestrasse 15, D-18119 Warnemünde, Germany.

# Corresponding Author: Carolina Reyes, creyes6@gmail.com

* Present address: University of Vienna, Department of Environmental Geosciences, Althanstrasse 14, 1090 Vienna, Austria. Telephone: +43-1-4277-53151

Key words: nitrogen, metagenome, marine, sediments

**Supporting Tables**

**Table S1.** Summary showing the number and types of sequences obtained after the MG-RAST pipeline analysis.

**Table S2.** Results of archaeal pyrosequencing results pertaining to Figure S1.

**Supporting Figures**

**Figure S1.** Archaeal diversity in SK and BB samples. Stacked bar graphs represent the abundance of major taxa in different samples relative to the total archaeal community. Relative abundances based on DNA 16S rRNA pyrosequences reported in Table S2.

**Supporting Methods**

*Pyrosequencing*

Details of the pyrosequencing method and analysis for the archaeal sequences reported in Figure S1 and Table S2 are found in Reyes and Noriega-Ortega, 2016 and references therein.

**Supporting References**

Reyes C, Noriega-Ortega BE. Report: Microbial diversity of Baltic Sea and North Sea sediments based on pyrosequencing results. FigShare doi: [10.6084/m9.figshare.3171442](https://dx.doi.org/10.6084/m9.figshare.3171442)
