# Supplementary material for: Nitrogen Metabolism Genes from Temperate Marine Sediments
Source: Mar Biotechnol (NY). 2017 Mar 10;19(2):175–90. doi: 10.1007/s10126-017-9741-0 (PMC5405112; doi:10.1007/s10126-017-9741-0)
Supplement: Supplementary file 2 — (DOCX 16 kb) [file 10126_2017_9741_MOESM2_ESM.docx]

Table S1. Summary showing the number and types of sequences obtained after the MG-RAST pipeline analysis.

| **Sample** | **BB34-F** | **BB34-R** | **BB67-F** | **BB67-R** | **SK68-F** | **SK68-R** |
| --- | --- | --- | --- | --- | --- | --- |
| MG-RAST ID | [4581509.3](http://metagenomics.anl.gov/linkin.cgi?metagenome=4581509.3) | [4581510.3](http://metagenomics.anl.gov/linkin.cgi?metagenome=4581510.3) | [4581512.3](http://metagenomics.anl.gov/linkin.cgi?metagenome=4581512.3) | [4581513.3](http://metagenomics.anl.gov/linkin.cgi?metagenome=4581513.3) | [4581515.3](http://metagenomics.anl.gov/linkin.cgi?metagenome=4581515.3) | [4581516.3](http://metagenomics.anl.gov/linkin.cgi?metagenome=4581516.3) |
| Depth (cmbsf)* | 3-4 cm | 3-4 cm | 6-7 cm | 6-7 cm | 6-8 cm | 6-8 cm |
| Number of raw sequences | 10,763,249 | 10,763,249 | 9,967,261 | 9,967,261 | 2,572,510 | 2,572,510 |
| Mean raw sequence length | 112 bp | 112 bp | 112 bp | 112 bp | 112 bp | 112 bp |
| Number of sequences as input | 8,718,851 | 8,718,851 | 8,322,592 | 8,322,592 | 2,156,253 | 2,156,253 |
| Mean sequence length | 97 ± 17 bp | 96 ± 18 bp | 95 ± 18 bp | 97 ± 17 bp | 100 ± 16 bp | 101 ± 16 bp |
| Number of sequences after MG-RAST quality control steps | 8,540,444 | 8,544,685 | 8,291,216 | 8,280,711 | 1,938,650 | 1,921,813 |
| Number of sequences with predicted protein features | 6,881,600 | 6,671,500 | 6,456,603 | 6,761,799 | 1,610,707 | 1,611,193 |
| Number of sequences with predicted rRNA features | 130,039 | 135,190 | 132,694 | 124,566 | 29,063 | 28,242 |
| Number of sequences with identified protein features | 1,203,618 | 1,151,500 | 991,177 | 1,079,686 | 209,364 | 216,761 |
| Number of sequences with identified rRNA features | 1,325 | 1,252 | 1,136 | 1,080 | 421 | 416 |
| Number of identified functional catagories | 910,117 | 871,246 | 735,362 | 805,438 | 152,179 | 158,111 |

* cmbf refers to centimeters below sea-floor

BB34 refers to sample 3-4 cmbsf , BB67 refers to sample 6-7 cmbsf, SK refers to sample 6-8 cmbsf

F and R refer to forward and reverse
