# Supplementary material for: Nitrogen Metabolism Genes from Temperate Marine Sediments
Source: Mar Biotechnol (NY). 2017 Mar 10;19(2):175–90. doi: 10.1007/s10126-017-9741-0 (PMC5405112; doi:10.1007/s10126-017-9741-0)
Supplement: Supplementary file 3 — (DOCX 15 kb) [file 10126_2017_9741_MOESM3_ESM.docx]

Table S2. Results of archaeal pyrosequencing results related to Figure S1.

| **Depth (cm)** | **Samples** | **Raw** | **Denoised** | **OTUs** | **Sequences taxonomically assigned as archaea** | **Avg. Sequence Length (bp)** | **DDBJ BioSample Accession Number** |
| --- | --- | --- | --- | --- | --- | --- | --- |
| 6-7 | BB67 | 10,662 | 8,035 | 145 | 5,211 | 222 | SAMD00031621 |
| 12-13 | BB1213 | 6,814 | 5,310 | 105 | 4,123 | 220 | SAMD00031620 |
| 21-26 | BB2126 | 7,587 | 5,795 | 113 | 4,265 | 224 | SAMD00031619 |
| **Total Sum** |  |  |  | **363** | **13,599** |  |  |
| 8-10 | SK810 | 38,520 | 26,605 | 1,298 | 16,204 | 220 | SAMD00031618 |
| 10-12 | SK1012 | 43,876 | 30,038 | 1,319 | 18,134 | 219 | SAMD00031617 |
| 16-23 | SK1623 | 29,518 | 20,259 | 852 | 15,346 | 215 | SAMD00031616 |
| **Total Sum** |  |  |  | **3,469** | **49,684** |  |  |
